# A Virtual Reality Force Control Training System on Brain Activation: Functional Near-Infrared Spectroscopy (fNIRS) Study

**DOI:** 10.2196/63874

**Published:** 2025-07-14

**Authors:** Luigi Gan, Chien-Ju Lin, Hsiao-Feng Chieh, Kai-Nan An, Fong-Chin Su

**Affiliations:** 1 Department of Biomedical Engineering, National Cheng Kung University, No. 1, Dasyue Rd, East District, Tainan, 701, Taiwan, 886 062757575 ext 63422; 2 Medical Device Innovation Center, National Cheng Kung University, Tainan, Taiwan; 3 Department of Orthopedic Surgery, Mayo Clinic, Rochester, MN, United States

**Keywords:** pinch force control, virtual reality, VR, functional near-infrared spectroscopy, aging, rehabilitation, exercises, exercise, elder, older adults, geriatric, neural plasticity, prefrontal cortex, PFC, premotor cortex, PMC, motor

## Abstract

**Background:**

Aging can bring upon several effects that can hinder one’s quality of life. One of the effects is the decline in one’s ability to perform activities of daily living, which is caused by the loss of hand function due to aging. To mitigate this, several virtual reality (VR)-based training or rehabilitation systems that use hand tracking were developed. Although these systems are effective, immersive, and can promote motivation, they are mostly limited to providing range of motion exercises. The addition of a force control component to the hand tracking of these systems could make them even more effective at improving or restoring hand function, as the majority of activities of daily living require a degree of force control.

**Objective:**

This study aimed to compare the effects of 2 VR input systems: regular hand tracking and the novel VR input system in this study, which incorporate force control to regular hand tracking on the brain activity of younger and older adults. The degree of cortical activity during a training or rehabilitation task is linked to better functional outcomes and improvements of neuroplasticity.

**Methods:**

Twelve younger adults (mean age 25.00, SD 4.50 years) and 12 older adults (mean age 73.00, SD 3.6 years) were recruited to play a game specifically developed for this study using 2 VR input systems. Brain activity during gameplay was recorded using functional near-infrared spectroscopy over the following cortical regions: prefrontal cortex (PFC), premotor cortex (PMC), supplementary motor area (SMA), and primary motor cortex (M1).

**Results:**

Compared with the regular hand-tracking system, adding a force control component increased average oxygenated hemoglobin (HbO) concentrations and decreased deoxygenated hemoglobin (HbR) concentrations in key brain regions. In young adults, these changes were observed in the right PMC and right M1. In older adults, higher HbO and lower HbR concentrations appeared in the right PFC, bilateral M1, and right SMA (HbR only). The force control component also led to more widespread activity across all ROIs.

**Conclusions:**

The novel input system in this study can be used for improving or restoring hand function. The results of this study can be used as a reference for the development of better VR-based training or rehabilitation systems.

## Introduction

Aging is a significant global issue, with the World Health Organization predicting that by the year 2030, 1 in 6 people will be aged 60 years or older [1]. These lead to impaired hand function, which can decrease one’s level of independence and ability to perform activities of daily living (ADLs).

Nowadays, the use of virtual reality (VR) as a platform for rehabilitation and training is becoming increasingly prevalent, as these systems can be effective [2-4] and are better at increasing one’s motivation [5] to undergo training or rehabilitation than conventional methods. This may be due in part to the platform’s ability to realistically simulate ADLs, reduce boredom and monotony compared with conventional methods, and provide user-adaptable difficulty levels [6,7]. Some notable examples of VR-based rehabilitation system that incorporate the VR features are the Amadeo hand rehabilitation device (Tyromotion GmbH) and the lab custom Tipr device. The Amadeo device is an interactive robotic device for hand function assessment, range of motion exercises, and force control rehabilitation. The Tipr, on the other hand, is a tabletop device specifically designed for older adult individuals and patients with mild cognitive impairment to engage in game-based interventions, using the forces from each finger as input. Trials for both devices show effectiveness in improving cognition, fine motor skills, and manual dexterity in users [8-11]. However, due to their tabletop design, such devices are limited to exercises where the hands remain fixed in a certain position during rehabilitation.

One of the advantages of using VR as a platform for providing rehabilitation and training is its flexibility in interactions methods, input systems, and feedback mechanisms, all of which can affect rehabilitation outcomes [12]. Among the various input systems for VR rehabilitation, hand tracking—where user’s hands are captured and displayed in the VR environment via cameras, allowing interaction through grasps, pokes, and so forth—is preferred by users with motor difficulties [13]. However, hand tracking alone limits the system to range of motion exercises. Adding a force control component could enhance these systems by making VR interactions more lifelike and similar to ADLs.

The opposable thumb, along with its ability to touch the other 4 fingers, makes human hands unique. Tasks involving the thumb and forefinger opposition, which accounts for a large portion of ADLs (dexterous manipulation of small objects, feeding, grooming, etc), are often accomplished by the precise control of pinch force of human fingers.

One method for evaluating the effects of different input systems, interaction methods, and game modes on upper extremity function [12,14,15], involves measuring brain activity using functional near-infrared spectroscopy (fNIRS). fNIRS is a noninvasive brain-imaging tool that assesses cortical activity by detecting changes in blood oxygen concentration in response to a stimulus. Higher brain activity during training or rehabilitation as indicated by changes in oxygenated (HbO) and deoxygenated hemoglobin (HbR) is associated with better functional outcomes and the promotion of neural plasticity [16-18].

This study aims to verify the effects of adding a force control component to a standard hand-tracking system on hand function recovery and improvement in younger adult and older adult populations. This is achieved by comparing the brain activity between the standard hand-tracking system (without force control [woFC]) and the novel hand-tracking system with force control (wFC) while playing a game developed for this study, with the hypothesis that playing the game wFC would elicit greater brain activity. The game simulates a feeding task, where the user must use a VR fork to pick up and deliver food to a baby. A more detailed explanation of the game is provided in the following sections.

## Methods

### Study Design and Setting

This study is a prospective, quantitative, and nonrandomized observational study examining the effects of 2 VR input systems (wFC and woFC) on brain activation in both younger adults and older adults. The research was conducted at the Motion Analysis Laboratory and the Medical Device Innovation Center of National Cheng Kung University (NCKU) in Tainan, Taiwan, from July 10, 2023, to August 11, 2023. The reporting adheres to the STROBE (Strengthening the Reporting of Observational Studies in Epidemiology) guidelines for observational research [19].

### Subject Recruitment

Participants were recruited through posters displayed around the NCKU campuses and local Line groups. The eligibility criteria included being at least 21 years of age, not having conditions that would prevent normal use of the VR system (such as finger or hand amputations), and not experiencing motion sickness when using the VR system.

### Ethical Considerations

The study was approved by the Human Research Ethics Review Committee of NCKU (approval NCKU HREC-E-112-181-2) and was also registered in ClinicalTrials.gov (NCT06412887). Informed consent was obtained from all participants before data collection began, including consent for the primary or secondary analysis and publication of research data. All data were kept anonymous. Participants received a compensation of US $18 upon completing the experiment.

### VR Force Control Training System—Hardware

**Figure 1.**
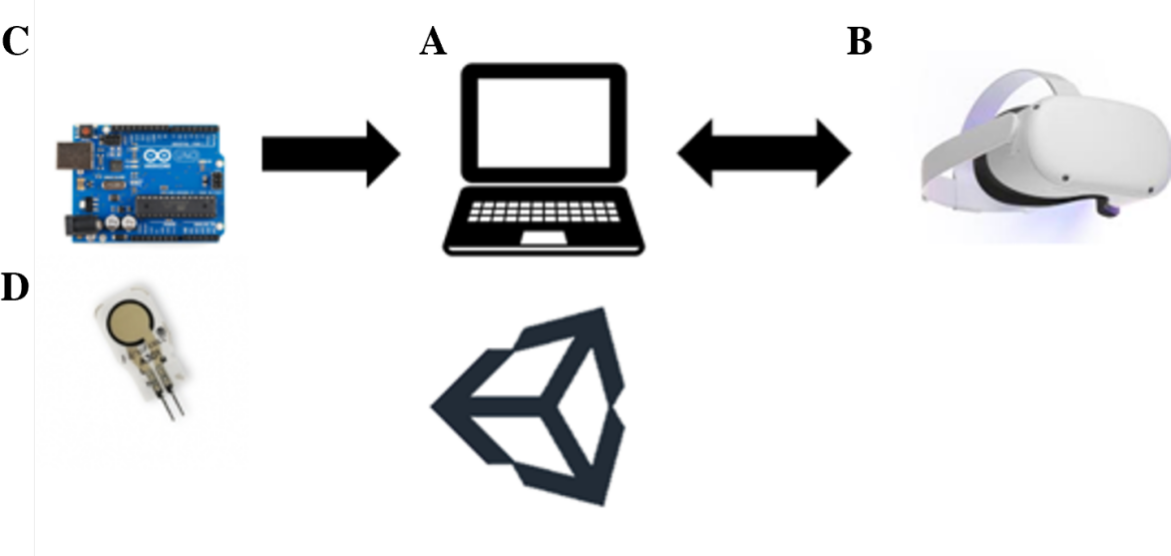
Hardware components of the virtual reality (VR) force control training system. (A) A laptop computer for running the VR game. (B) Meta Quest 2 head-mounted display for displaying the game and capturing the position and poses of the user's hands. (C) Arduino Uno for detecting, converting, and sending the changes in the resistance values of the force-sensitive resistor (FSR) to the laptop computer running Unity. (D) FSR for capturing and measuring changes in the pressure and force exerted between the thumb and the index finger.

The hardware part of the VR Force Control Training System consists of 4 main components. A laptop computer running Unity (Unity Technologies) is labeled A in Figure 1. A head-mounted display, specifically the Meta Quest 2 (Meta Platforms Inc), is labeled B in Figure 1 and is used to display the game. A force-sensing system, built around a Flexiforce A301 force-sensitive resistor (FSR) from Tekscan, Inc, is labeled D in Figure 1. This FSR was calibrated using test weights ranging from 300 g to 7 kg with 5 trials per weight, each lasting 10 seconds. An Arduino Uno, labeled C in Figure 1, converts and sends the FSR readings to the laptop computer running Unity. Figure 1 illustrates the VR force control training system used in this study. The system’s stability was previously evaluated by measuring 3 different test weights over 3 days at 3-hour time intervals. The resulting intraclass correlation coefficient over 3 days was 0.75.

### VR Force Control Training System—Software

As mentioned in the previous sections, the game consists of 2 tasks that can be played using 2 input systems, both based on the hand-tracking features provided by the Oculus Interaction SDK. The first task requires users to memorize a 5-element list containing the order of the food to be fed to the baby. The second task involves picking up an interactive fork, selecting the appropriate food, and feeding it to the baby. In the woFC system, the user simply performs a thumb-index finger opposition to pick up the fork. In contrast, the wFC system is designed with the same thumb-index finger opposition in woFC system but with an additional isometric pinch contraction at 70 (SD 10%) maximum voluntary contraction (MVC), a value derived from a prior pilot study.

**Figure 2.**
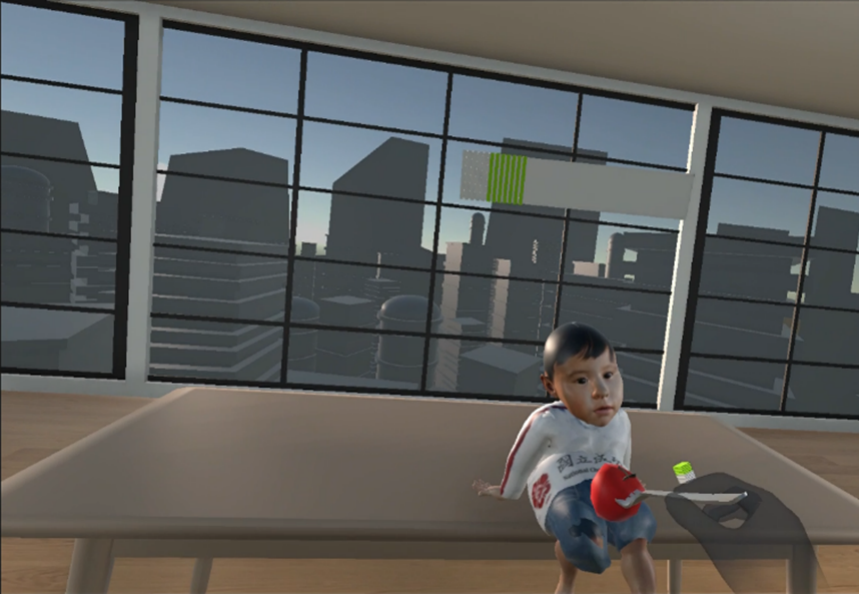
A user maintaining the pinch force at the allowable range, represented by the green area of the force bar, going above or below the allowable range displays the red and white areas, respectively.

**Figure 3.**
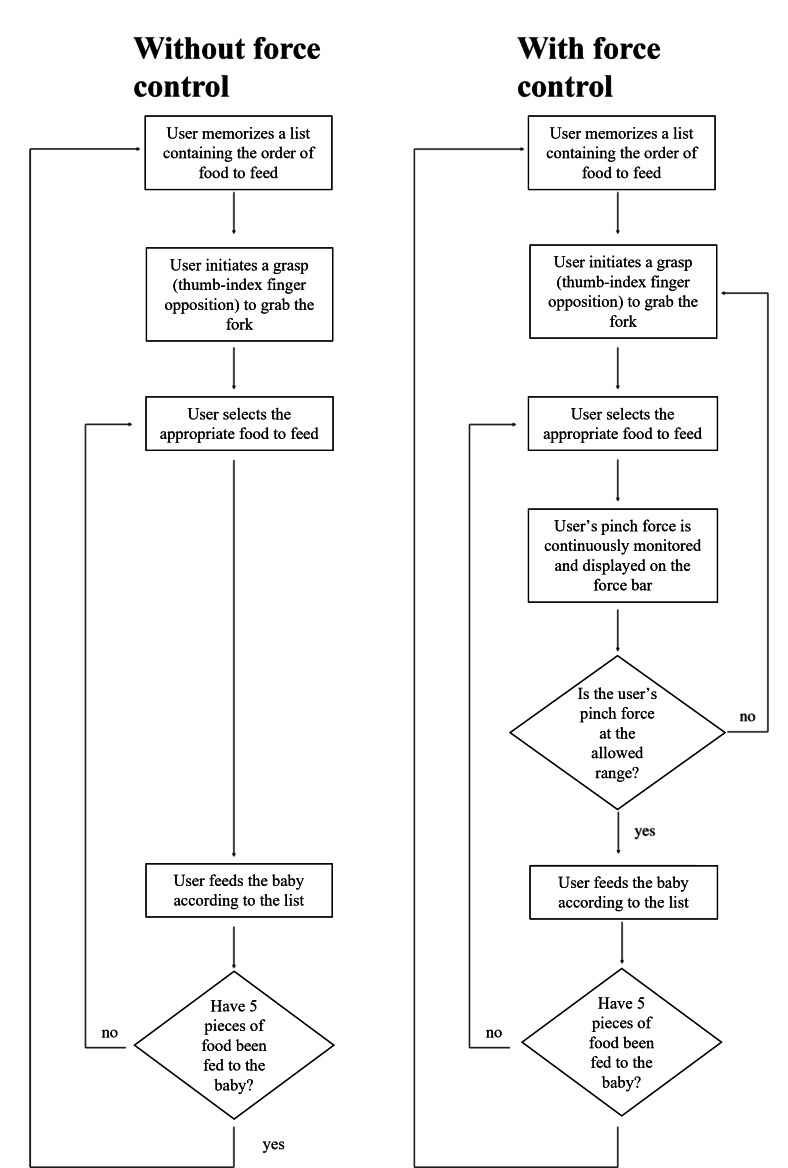
Flowchart illustrating the differences between without force control (left) and with force control (right).

Once the fork is picked up in the wFC system, the user’s pinch force is continuously monitored and displayed on a force bar and provides real-time feedback as depicted in Figure 2. Exceeding or falling below the allowable pinch force range causes the fork to reset, requiring the users to initiate the grasp again. These modifications in the wFC system introduce an added layer of difficulty immersion compared with the woFC system. To achieve this functionality, custom code was written to modify the hand-tracking features of the Oculus Interaction SDK. Differences between wFC and woFC systems are summarized by Figure 3.

### Experiment Setup and Procedures

**Figure 4.**
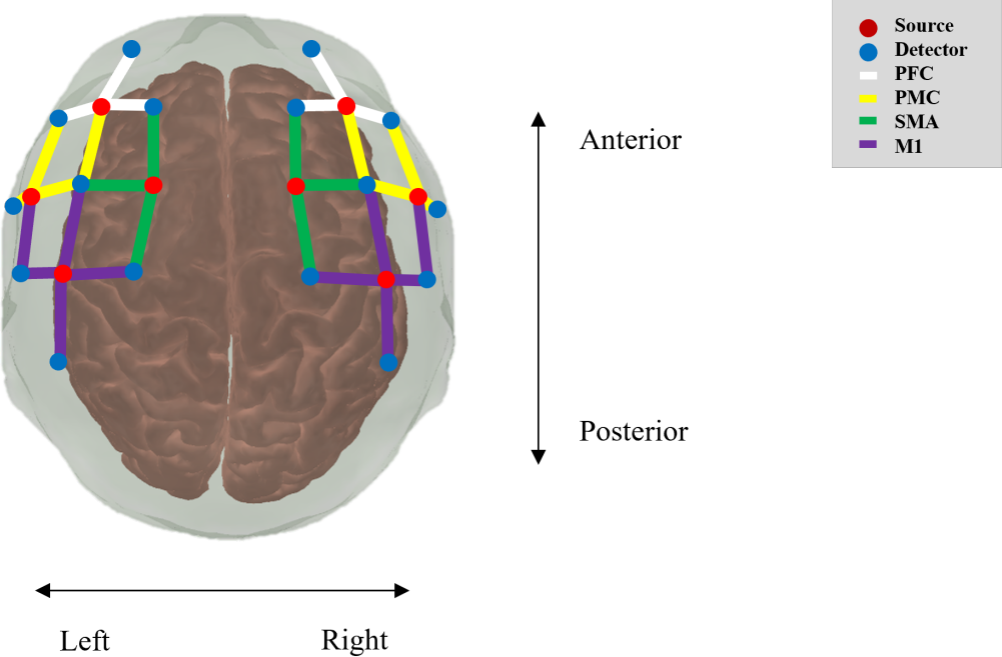
Arrangement of the source and detectors optodes for this study. Source and detector optodes are represented by red and blue dots, respectively. Regions of interest are formed from multiple color-coded channels with the PFC, PMC, SMA, and M1 formed by white, yellow, green, and violet channels respectively. M1: primary motor cortex; PFC: prefrontal cortex; PMC: premotor cortex; SMA: supplementary motor area.

**Figure 5.**
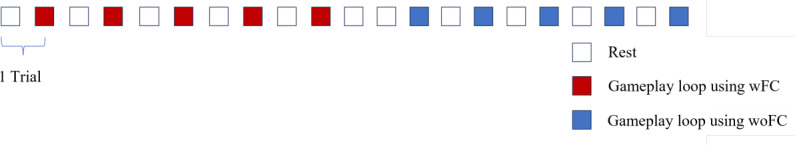
The event-related paradigm used in this study. Rest, gameplay loop using wFC, and gameplay loop using woFC are represented by white, red, and blue squares, respectively. wFC: with force control; woFC: without force control.

Brain activity was measured using NIRScout system (NIRx Medical Technologies). The setup consisted of 8 sources and 16 detector optodes, spaced 3 cm apart, forming 30 channels. These were arranged according to the international 10‐20 system Figure 4 to cover the regions of interest (ROIs) involved with hand movements, specifically the prefrontal cortex (PFC), premotor cortex (PMC), supplementary motor area (SMA), and primary motor cortex (M1). Data were collected over 2 wavelengths (760 nm and 850 nm) with a sampling rate of 7.81 Hz. The experiment followed a block-related design as illustrated in Figure 5. A trial in this study consisted of a single gameplay loop, followed by a rest event, each lasting 30 seconds. Measurements were taken during 5 consecutive trials using the gameplay loop wFC, followed by a rest event and then 5 consecutive trials using the gameplay loop woFC. During each gameplay loop the participants played the game using the assigned input system. In contrast, during the rest events, they were asked to close their eyes and enter a resting state.

**Figure 6.**
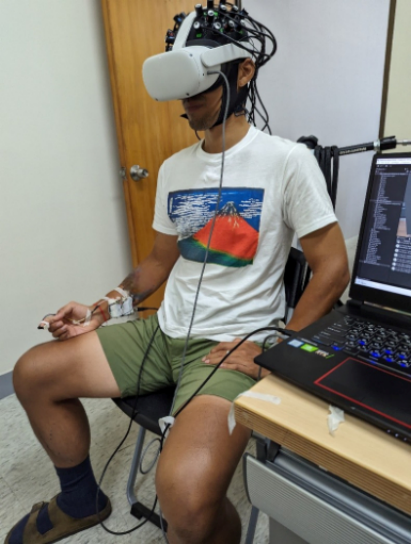
A participant playing the game using the wFC (with force control).

The experiment procedures were as follows: First, participants entered an isolated room where they were briefed about the experiment and provided informed consent. Second, participants familiarized themselves with both input systems of the game, and the in-game maximum pinch voluntary contraction was recorded to calculate each user’s force threshold. Third, the fNIRS system was calibrated using the NIRStar software (NIRx Medical Technologies) to ensure acceptable signal quality across all channels. Finally, data collection proceeded according to the block-related design outlined in Figure 5, while Figure 6 shows a participant performing the experiment.

### Data Processing and Analysis

Game performance was quantified using 3 parameters: the amount of grasp initiated, pinch force coefficient of variation (COV), and memory task accuracy. As the amount of grasp initiated, a unitless metric was derived by counting the number of times the participants needed to pick up the fork in each gameplay loop, reflecting the number of times the participants’ pinch force exceeded or fell below the threshold. The pinch force COV was calculated by dividing the SD of the pinch force by the average pinch force during each gameplay loop, resulting in a unit of Newton/Newton. Memory task accuracy was calculated by dividing the number of correct foods fed by the total number of foods fed in each gameplay loop.

Raw fNIRS signals were processed using MATLAB R2023b (Mathworks) running the open-source app Homer3 (version 1.80.2) [20]. The signals were preprocessed manually and by using functions inside Homer3. These include visually inspecting the raw signals for noise and discontinuities, channel pruning, conversion from intensity to optical density, motion corrections, bandpass filtering, and conversion from optical density to concentration. Channel pruning was performed to eliminate signals that were too weak, too strong, or had a high SD. Motion correction was done using the Wavelet-based algorithms [21]. Bandpass filter was set at 0.01 Hz and 0.1 Hz was applied to eliminate the effects of heartbeat, breathing, and other Mayer waves [22]. The optical densities were then converted into concentration using the modified Beer-Lambert Law with the default partial path length factor of 1.

The hemodynamic response function (HRF) of HbO and HbR was estimated with general linear modeling using the ordinary least squares method [23]. The HRF was modeled by a consecutive sequence of Gaussian functions, with SD of 1 and a temporal spacing of 1 second between the mean over a period of −5 to 35 seconds [24]. The baseline period was set to 5 seconds before stimulus onset, and its mean was subtracted from the whole HRF (−5 seconds to 35 seconds) to perform baseline normalization. The mean HbO and HbR concentration for each ROI was calculated by averaging across the channels in each ROI from the period of 10 seconds to 35 seconds owing to the delay in movement-induced hemodynamic response [22]. The mean HbO and HbR concentration was then projected to the cortical surface of the Colin Atlas by registering the optode and channel locations in AtlasViewer (version 2.44) [25], a MATLAB toolbox for the anatomical interpretation of fNIRS data processed in Homer3.

The Pearson correlation between each ROI’s processed HbO and HbR data was calculated for each group, resulting in four 8×8 matrices representing 4 full network connections. For a more comprehensive evaluation of the network, 2 features were extracted for statistical analysis: connection ratio (CR) and connection strength (CS) [26]. To obtain CR and CS, the Pearson correlation coefficient of each matrix was first converted to normal distribution through the Fisher *z* transformation [27]. CR was defined as the ratio between the significant connections and the total number of connections in each network, while CS was defined as the average of absolute *z* values of a network. A connection was considered significant when its absolute *z* value was greater than 0.7.

The Mann-Whitney *U* test was used for comparing the game performance between younger adult and older adult populations. The Wilcoxon signed rank test was used to assess the statistical significance of differences in HbO and HbR responses, CS, and CR between the 2 input systems. The Spearman correlation was used to identify correlations between game scores and HbO and HbR responses. The level of significance was set at .05. For data involving multiple comparisons, the Benjamini-Hochberg procedure [28] was used to control the false discovery rate with a Benjamini-Hochberg critical value of 0.05.

## Results

This section aims to present the main results of this study, namely, game performance, mean HbO concentration, temporal characteristics of HRF, and the cortical activation maps.

### Participants

The study included 12 eligible younger adults (mean age 25.00, SD 4.5 years) and 12 eligible older adults (mean age 73.00, SD 3.6 years). Of the participants, 23 were right-handed; the dominant hand of 1 participant was not specified.

### Game Performance

Table 1 shows the average game scores per run and its comparison between both groups. Only the pinch force COV showed a statistically significant difference.

**Table 1.** Game performance of both groups.

	Younger adults	Older adults	*P* value
Amount of grasp initiated^a^, mean (SD)	5.02 (3.13)	5.26 (2.79)	.67
Pinch force coefficient of variation^b^, mean (SD)	1.23 (0.40)	1.6 (0.98)	.03
Memory task accuracy^c^, mean (SD)	0.83 (0.12)	0.72 (0.23)	.11

a Amount of grasp initiated is a unitless metric.

b Pinch force coefficient of variation is in the unit of Newton/Newton.

c Memory task accuracy is a unitless metric.

### Mean HbO Concentration

The mean HbO concentration for the younger adults and the older adults during gameplay is shown in Tables2  3, respectively. Some ROIs show significantly higher HbO concentration when playing the game using the wFC, namely, the right premotor cortex (rPMC); right primary motor cortex (rM1) for the younger adults; and left primary motor cortex (lM1), right prefrontal cortex (rPFC), and rM1 for the older adults.

**Table 2.** Mean values of oxygenated hemoglobin concentration for the younger adult group under while playing the game both with force control and without force control input systems^a^.

Younger adult group	lPFC^b^	lPMC^c^	lSMA^d^	lM1^e^	rPFC^f^	rPMC^g^	rSMA^h^	rM1^i^
wFC^j^ HbO^k^ mean	32.88 (9.49)	70.83 (20.45)	39.51 (15.49)	53.66 (15.49)	41.73 (12.05)	81.14 (23.42)	16.62 (4.80)	35.15 (10.15)
woFC^l^ HbO mean	23.18 (6.69)	33.66 (9.72)	26.82 (7.74)	26.97 (7.78)	25.67 (7.41)	16.60 (4.79)	1.48 (0.43)	−0.44 (0.13)
*P* value	.583	.034	.272	.050	.182	.003	.084	.003
Adjusted *P* value	.583	.907	.312	.100	.243	.012	.134	.012

a Data are presented in mean (SE) of the mean. The prefixes l and r stand for left and right, respectively. Units are in μM-mm

b lPFC: left prefrontal cortex.

c lPMC: left premotor cortex.

d lSMA: left supplementary motor area.

e lM1: left primary motor cortex.

f rPFC: right prefrontal cortex.

g rPMC: right premotor cortex.

h rSMA: right supplementary motor area.

i rM1: right primary motor cortex.

j wFC: with force control.

k HbO: oxygenated hemoglobin.

l woFC: without force control.

**Table 3.** Mean values of oxygenated hemoglobin concentration for the older adult group while playing the game under both with force control and without force control input systems^a^.

Older adult group	lPFC^b^	lPMC^c^	lSMA^d^	lM1^e^	rPFC^f^	rPMC^g^	rSMA^h^	rM1^i^
wFC^j^ HbO^k^ mean	38.58 (11.14)	41.67 (12.03)	51.99 (15.01)	62.63 (18.08)	37.89 (10.94)	45.19 (13.05)	58.81 (16.98)	59.56 (17.19)
woFC^l^ HbO mean	25.17 (7.27)	33.48 (9.66)	48.35 (13.96)	42.37 (12.23)	16.51 (4.77)	30.13 (8.70)	35.92 (10.37)	37.84 (10.92)
*P* value	.158	.875	.583	.015	.015	.272	.041	.012
Adjusted *P* value	.253	.875	.666	.040	.040	.363	.082	.040

a Data are presented in mean (SE) of the mean. The prefixes l and r stand for left and right, respectively. Units are in μM-mm.

b lPFC: left prefrontal cortex.

c lPMC: left premotor cortex.

d lSMA: left supplementary motor area.

e lM1: left primary motor cortex.

f rPFC: right prefrontal cortex.

g rPMC: right premotor cortex.

h rSMA: right supplementary motor area.

i rM1: right primary motor cortex.

j wFC: with force control.

k HbO: oxygenated hemoglobin.

l woFC: without force control.

### Mean HbR Concentration

The mean HbR concentration for the younger adults and the older adults during gameplay is shown in Tables4  5, respectively. Some ROIs show significantly lower HbR concentration when playing the game using the wFC, namely, the rPMC; rM1 for the younger adults; and lM1, rPFC, right supplementary motor area (rSMA), and rM1 for the older adults.

**Table 4.** Mean values of deoxygenated hemoglobin concentration for the younger adult group while playing the game under both with force control and without force control input systems^a^.

	lPFC^b^	lPMC^c^	lSMA^d^	lM1^e^	rPFC^f^	rPMC^g^	rSMA^h^	rM1^i^
wFC^j^ HbR^k^ mean	−19.85 (5.73)	−42.72 (12.33)	−29.06 (8.39)	−29.77 (8.59)	−18.05 (5.21)	−51.11 (14.75)	−4.77 (1.38)	−18.36 (5.30)
woFC^l^ HbR mean	−9.73 (2.81)	−20.06 (5.79)	−20.23 (5.84)	−14.45 (4.17)	−10.42 (3.01)	−12.83 (3.70)	4.19 (1.21)	−0.96 (0.28)
*P* value	.239	.023	.308	.060	.182	.003	.117	.004
Adjusted *P* value	.273	.062	.308	.120	.243	.016	.187	.016

a Data are presented in mean (SE) of the mean. The prefixes l and r stand for left and right, respectively. Units are in μM-mm.

b lPFC: left prefrontal cortex.

c lPMC: left premotor cortex.

d lSMA: left supplementary motor area.

e lM1: left primary motor cortex.

f rPFC: right prefrontal cortex.

g rPMC:right premotor cortex.

h rSMA: right supplementary motor area.

i rM1: right primary motor cortex.

j wFC: with force control.

k HbR: deoxygenated hemoglobin.

l woFC: without force control.

**Table 5.** Mean values of deoxygenated hemoglobin concentration for the older adult group while playing the game under both with force control and without force control input systems^a^.

	lPFC^b^	lPMC^c^	lSMA^d^	lM1^e^	rPFC^f^	rPMC^g^	rSMA^h^	rM1^i^
wFC^j^ HbR^k^ mean	−14.53 (4.19)	−21.96 (6.34)	−19.83 (5.72)	−21.83 (6.30)	−12.60 (3.64)	−23.07 (6.66)	−17.68 (5.10)	−19.10 (5.51)
woFC^l^ HbR mean	−8.84 (2.55)	−16.17 (4.67)	−19.78 (5.71)	−14.52 (4.19)	−4.03 (1.16)	−13.56 (3.92)	−9.16 (2.65)	−12.12 (3.50)
*P* value	.158	.638	.875	.023	.015	.308	.010	.008
Adjusted *P* value	.253	.729	.875	.046	.040	.411	.040	.040

a Data are presented in mean (SE) of the mean. The prefixes l and r stand for left and right, respectively. Units are in μM-mm.

b lPFC: left prefrontal cortex.

c lPMC: left premotor cortex.

d lSMA: left supplementary motor area.

e lM1: left primary motor cortex.

f rPFC: right prefrontal cortex.

g rPMC: right premotor cortex.

h rSMA: right supplementary motor area.

i rM1: right primary motor cortex.

j wFC: with force control.

k HbR: deoxygenated hemoglobin.

l woFC: without force control.

### Correlation Matrices Between ROIs

**Figure 7.**
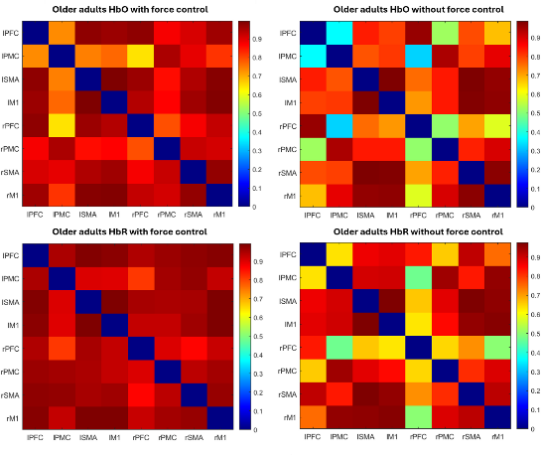
HbO (top) and HbR (bottom) correlation matrices of the older adults during with force control (left) and without force control (right) gameplay. HbO: oxygenated hemoglobin; HbR: deoxygenated hemoglobin; IM1: left primary motor cortex; IPFC: left prefrontal cortex; IPMC: left premotor cortex; ISMA: left supplementary motor area; rM1: right primary motor cortex; rPFC: right prefrontal cortex.rPMC: right premotor cortex.rSMA: right supplementary motor area.

**Figure 8.**
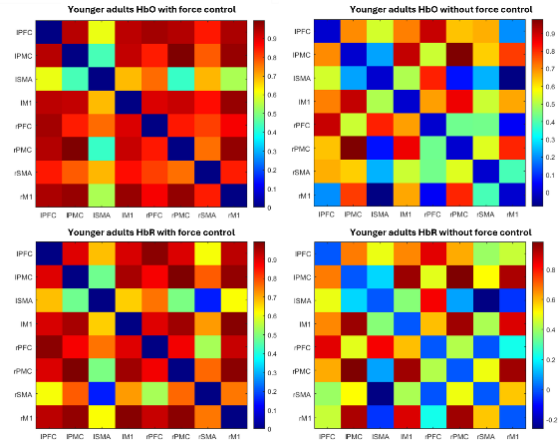
HbO (top) and HbR (bottom) correlation matrices of the younger adults during with force control (left) and without force control (right) gameplay. HbO: oxygenated hemoglobin; HbR: deoxygenated hemoglobin; IM1: left primary motor cortex; IPFC: left prefrontal cortex; IPMC: left premotor cortex; ISMA: left supplementary motor area; rM1: right primary motor cortex; rPFC: right prefrontal cortex.rPMC: right premotor cortex.rSMA: right supplementary motor area.

The correlation matrices between the HbO and HbR data of each ROI for both groups, while playing the game with and without force control (wFC and woFC), are presented in Figures 7 and 8, respectively. ROIs with higher correlations appear in reddish colors, while ROIs with less correlations appear in bluish colors.

### Connection Strength and Connection Ratio

The CS and CR for the younger adults and the older adults are shown in Table 6. The older adult group exhibited higher CS and CR when playing the game using wFC as compared with woFC.

**Table 6.** Network features of both groups while playing the game under with force control and without force control input systems^a^.

	Older adult		Younger adult	
	Connection strength	Connection ratio	Connection strength	Connection ratio
wFC^b^	1.15 (0.33)	0.76 (0.22)	0.79 (0.23)	0.48 (0.14)
woFC^c^	0.94 (0.27)	0.62 (0.18)	0.69 (0.20)	0.43 (0.13)
*P* value	.010	.018	.117	.373

a Data are presented in mean (SE) of the mean.

b wFC: with force control.

c woFC: without force control.

### Temporal Characteristics of HRF

**Figure 9.**
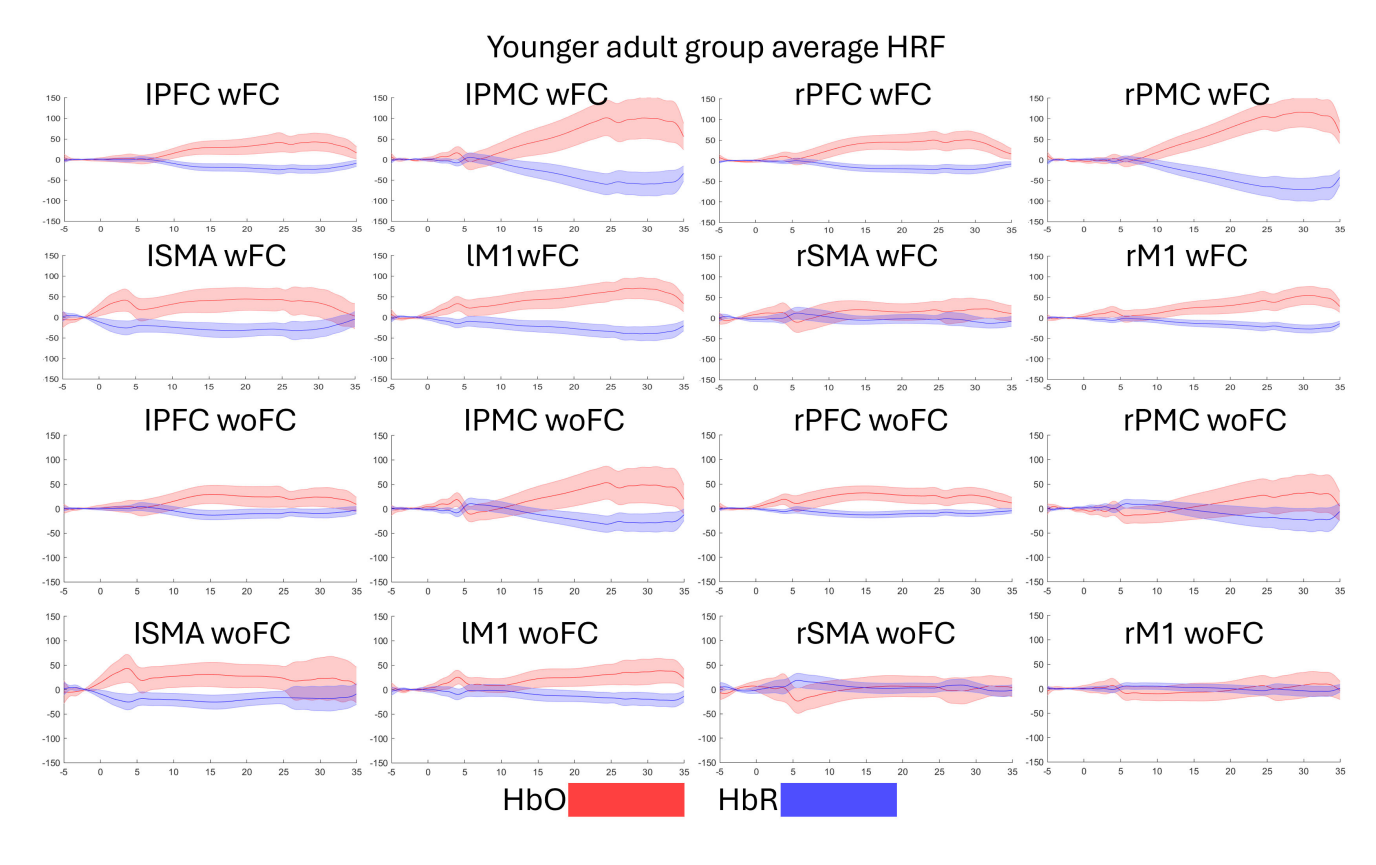
Temporal characteristics of the HRF of the younger adult group. Red represents the HbO, while blue represents the HbR. The shaded regions are the SE of mean. The figure is divided into the HRF between the left (first to second columns) and right (third to fourth columns) hemispheres and between wFC (first to second rows) and woFC (third to fourth rows). The *x*-axis represents time in seconds, while the *y*-axis represents HRF concentration in μM-mm. HbO: oxygenated hemoglobin; HbR: deoxygenated hemoglobin; HRF: hemodynamic response function; lM1: left primary motor cortex.; lPFC: left prefrontal cortex; lPMC: left premotor cortex; lSMA: left supplementary motor area; PFC: prefrontal cortex; rm1: right primary motor cortex; rPMC: right premotor cortex; rSMA: right supplementary motor area; wFC: with force control; woFC: without force control.

**Figure 10.**
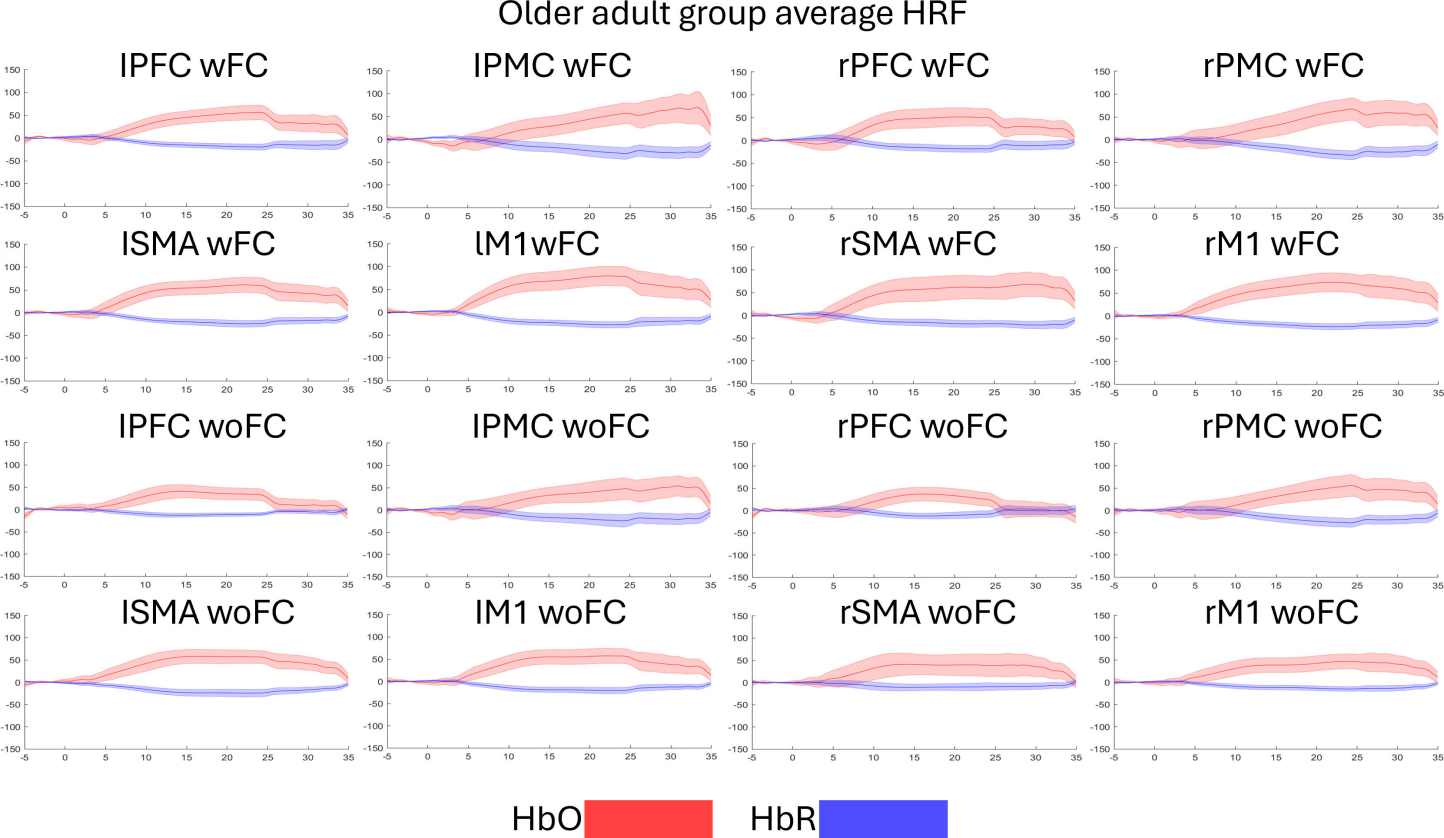
Temporal characteristics of the HRF of the older adult group. Red represents the HbO, while blue represents the HbR. The shaded regions are the SE of mean. The figure is divided into the HRF between the left (first to second columns) and right (third to fourth columns) hemispheres and between wFC (first to second rows) and woFC (third to fourth rows). The *x*-axis represents time in seconds, while the *y*-axis represents HRF concentration in μM-mm.HbO: oxygenated hemoglobin; HbR: deoxygenated hemoglobin; HRF: hemodynamic response function; lM1: left primary motor cortex.; lPFC: left prefrontal cortex; lPMC: left premotor cortex; lSMA: left supplementary motor area; PFC: prefrontal cortex; rm1: right primary motor cortex; rPMC: right premotor cortex; rSMA: right supplementary motor area; wFC: with force control; woFC: without force control.

Figures 9 and 10 present the temporal characteristics of the HRF (HbO and HbR) of both groups during gameplay. In Figure 9, the HbO temporal characteristics of the PFC, PMC, and M1 during wFC follow a rise after task onset that slowly decreases as the task progresses. The HbR follows the opposite pattern. As for the SMA, the left side, regardless of input system, shows a spike in the HbO and the HbR, followed by a constantly elevated response, while the HbO and the HbR of the right side stay at around baseline levels. Regarding the M1 during woFC, the HbO and the HbR on both the left and right sides show little to no response. For the older adult group, Figure 10 shows that all ROIs follow an increase in HbO after task onset, which slowly decreases as the task progresses, while the HbR follows an opposite pattern.

### Cortical Activation Maps

**Figure 11.**
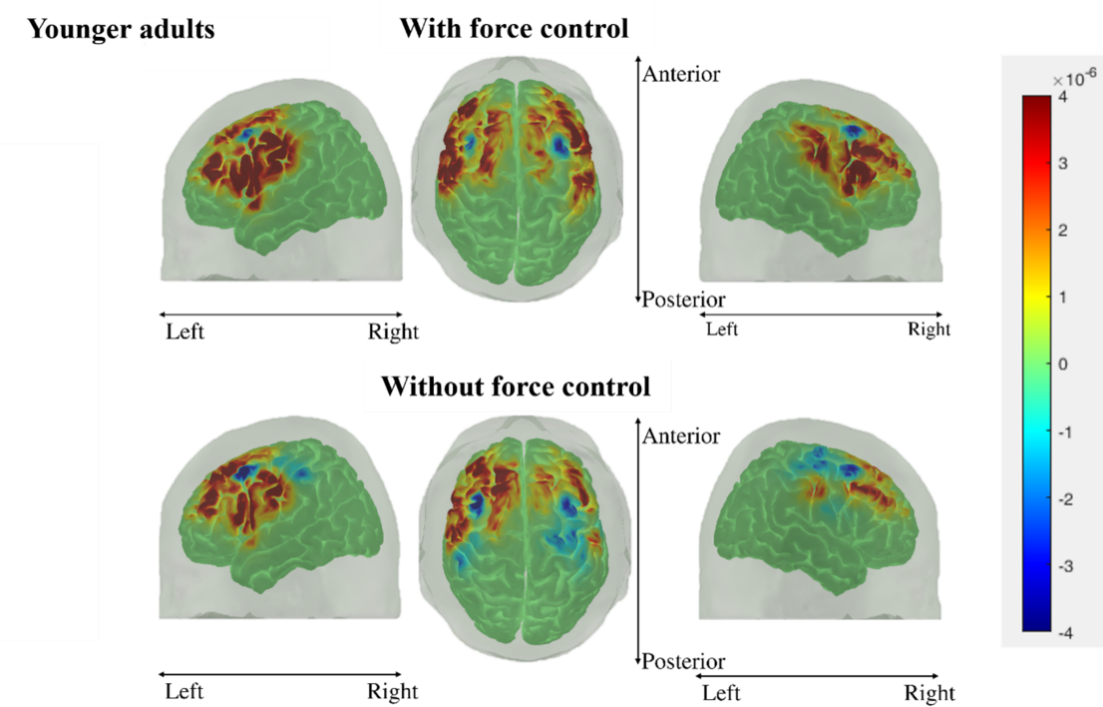
Cortical activation maps of the younger adult group during gameplay using both input systems. Top 3 images are the cortical activation maps during gameplay using with force control, while the bottom 3 images are the cortical activation maps during gameplay using without force control.

**Figure 12.**
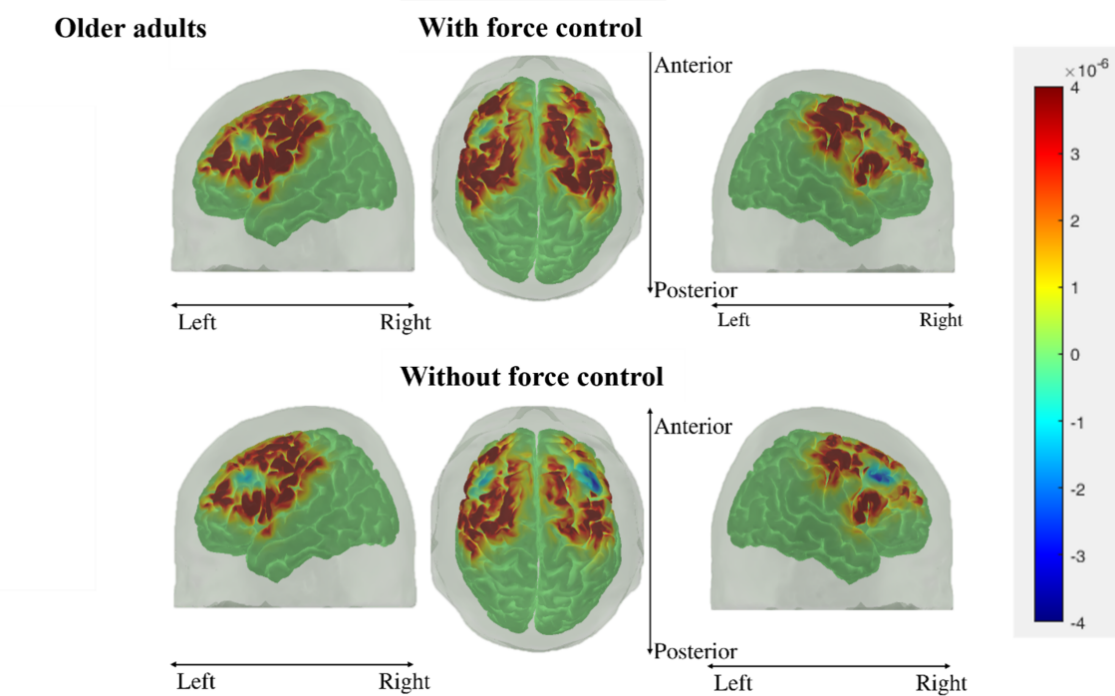
Cortical activation maps of the older adult group during gameplay using both input systems. Top 3 images are the cortical activation maps during gameplay using with force control, while the bottom 3 images are the cortical activation maps during gameplay using without force control.

Figures 11 and 12 show the cortical activation maps during gameplay under both input systems for the younger adult and older adult groups, respectively. The red color signifies increases in HbO while blue signifies decreases in HbO. Based on both figures, the wFC elicits a larger area of activation than the woFC, irrespective of age groups. Age-related differences occur in the laterality of activation, with the older adults experiencing a more bilateral activation across 2 input systems.

## Discussion

### Principal Findings

This study investigated the effects of integrating force control into a VR hand-tracking system on brain activity in the younger adults and the older adults. Participants played the same game using 2 different control systems (wFC and woFC). Despite studies showing that there is a clear difference in game performance between younger adults and older adults, whether it be commercial games [29], exergames [30,31], cognitive games [32], and so forth, the results of this study did not (amount of grasp initiated and memory task accuracy). In the aforementioned studies, aging-related changes and the level of difficulty were identified as the main factors for the differences in game performance. As for this study, the difficulty influenced by the force threshold and number of elements in the food list could not have been challenging enough, which resulted in similar performance across age groups.

### Comparison to Prior Work

The findings of this study align with previous fNIRS research [12,33] on finger opposition movement as a primary input and the effects of different interaction modes in both immersive and nonimmersive VR games. These studies suggest that changes in interaction mode or input system can influence movement patterns, posture, perceived difficulty, and enjoyment, all of which may contribute to variations in brain activity intensity and the areas of activated brain regions. Looking further into how the 2 input systems (wFC and woFC) affected each ROIs reveals the following:

First, the addition of force control can introduce elements that can alter the movement strategies of the participants as reflected by the higher right PMC activity in the younger adults and higher rSMA activity in the older adults during wFC gameplay. Aside from motor planning, the PMC is also responsible for action observation and transforming visual information into motor commands. This includes the anticipation of the force output required to lift an object based on visual cues [34]. Furthermore, the precision of one’s pinch force control can also greatly affect the PMC activation, with tasks requiring a higher precision of pinch force control, such as holding a small object with a sustained force level of just above the point before the object slips, producing a much higher right PMC activity as compared with holding the small object with one’s normal and automatically scaled pinch force [35]. The right PMC activity of the younger adult group in this study also followed the behavior found in the previous studies. The lack of difference in the right PMC activation of the older adults when playing the game in both control systems can be due to their right PMC already being recruited to play the game using the woFC as a compensatory mechanism by the aging brain to recruit additional cortical areas when performing simple motor tasks [36,37]. Furthermore, this mechanism, along with the wFC’s requirement to follow a precise movement sequence (thumb and index finger opposition followed by sustained isometric contraction at a strict time window ~0.5 second), likely contributed to the higher rSMA activity exhibited by the older adults, as well as the weak to moderate correlation between the pinch force COV-HbO (*r*=0.393) and pinch force COV-HbR (*r*=−0.494). The SMA is directly involved in planning complex movements that require temporal accuracies, as revealed by studies requiring users to press a button 2.5 seconds after a stimulus [38-40] and in studies disrupting the function of the SMA via repetitive transcranial magnetic stimulation, revealing worse performance in a sequential pressing task.

Second gameplay using the wFC induces greater ipsilateral M1 activity in both groups than gameplay using the woFC. In the young and healthy population, simple movements involving the hands and fingers exclusively activate the contralateral M1, while older adults feature more bilateral activity in the motor regions in response to motor tasks [36,41-43]. Aside from being age dependent, the ipsilateral activity of the M1 could also be influenced by task requirements and complexity [44-47]. Tasks that require sustained isometric contraction can also induce bilateral activity in the M1, detectable by both fNIRS and functional magnetic resonance imaging (fMRI) [48-50]. The magnitude of the force during these tasks ranges from 20% to 80% MVC and lasts from 10 up to 180 seconds per trial. Results from these studies point out that the bilateral activity of the M1 can be achieved by finger tasks accompanied by 20%, 40%, and 60% MVC. The contralateral activity of the M1 does not scale directly with force output, and the ipsilateral M1 is directly affected by the magnitude of the force, as well as the duration of the muscle contraction. This study also obtained consistent results with the previously mentioned studies. The results of this study aligning with previous findings show that wFC gameplay induces higher rM1 activity than woFC gameplay in both groups. This can be attributed to the wFC requiring users to exert an isometric contraction at approximately 70% MVC and maintain it for a few seconds to play the game effectively. Additionally, a positive correlation was observed between pinch force COV and HbO, with higher pinch force COV scores being associated with greater rM1 activity (*r*=0.494). One thing to take note of is that only the older adults exhibited higher activity in the lM1 in addition to the rM1 during gameplay using the wFC as compared with the woFC. This finding is also consistent with the previous studies mentioned, where older adults generally exhibit more bilateral activation in the motor regions than younger adults performing the same task.

Finally, rPFC in the older adults is recruited to keep up with the higher demands of playing the VR game when using the wFC. Aside from being involved with cognitive functions [51,52] and motor tasks [53], numerous studies also link increasing attentional demands, dual tasking costs, task difficulty, and complexity to the degree of PFC activity [54-57]. The functions of the PFC can also be divided further between the left and right hemispheres, with the rPFC being implicated for both externally and internally cued [58] real-time monitoring of movement and for the retrieval of memory [59,60]. As for this study, in addition to the memory and motor tasks when using the woFC, the introduction of a force control task adds a layer of complexity to the game when using the wFC. This could have caused the older adult group to allocate more attentional and memory resources for adjusting their force output through monitoring of the force bar, as reflected by the similar number of grasps initiated and the higher pinch force COV as compared with the younger adults, while at the same time recalling the order of the food list. fMRI studies [61,62] also found rPFC activity during a precision force control task and cited the visual feedback as a contributing factor to the rPFC activity.

Stronger CS and CR in previous studies reflect an increase in coordinating the senses and motor control function [63] and an additional cognitive load [64] in response to task complexity. In this study’s wFC, the additional requirement of maintaining one’s pinch force, alongside the compensatory mechanism of the aging brain mentioned in the previous sections, may explain higher CS and CR in the older adults during wFC.

The temporal characteristics of the HRF for both groups tend to follow the typical hemodynamic response as those found in the literature [14,17] in which activated brain regions experienced a rise in the concentration of HbO, accompanied by a drop in the concentration of HbR.

When considering the cortical activation maps, differences can be observed between the 2 input systems and between the 2 study groups. Comparing the cortical activation maps of the 2 input systems shows that the wFC activates a larger spatial activity in the brain. fMRI and positron emission tomography studies [50,65-69] have also established a link between the extent of the spatial activity of the brain to the force level and the presence of an isometric contraction during a handgrip task. These studies suggest that recruiting larger areas of the cortex is an adaptation mechanism by the brain for sustaining isometric force tasks. The same process could have also occurred in this study, as gameplay using the wFC requires an isometric pinch as opposed to the woFC system where closing the gap between the index finger and the thumb is enough to play the game properly. On the other hand, a comparison of the cortical activation maps between the 2 study groups reveals that the older adult group experienced more bilateral brain activity irrespective of control systems. fMRI studies [43,70], where subjects were asked to perform finger opposition and hand movements, also obtained comparable results with their older adult groups exhibiting a more bilateral activity in several cortical regions (PMC, SMA, M1, etc) than their younger adult counterparts. The effects of aging on the corpus callosum are cited to be one of the culprits to the reduction of hemispheric lateralization in the older adults.

### Limitations and Future Directions

This study is limited by the following factors: First, the study population consisted of only healthy individuals, so the effects of the developed system on the Hb response of patients with neurological symptoms cannot be determined. Second, the experiment used a fixed block design—5 consecutive wFC gameplay sessions followed by 5 consecutive woFC sessions—to minimize the time interval between conditions. This design was chosen to reduce delays, as removing the sensor was quicker than reapplying it midexperiment, which could have introduced systematic bias. Third, the single game difficulty level was used in the study, as different game difficulty levels [71] can directly affect the Hb responses of the brain. Fourth, the long-term training or rehabilitation effects of this system cannot be determined in this study as this study was a one-time intervention. Fifth, the usage of a single sensor in the system, although transposable, limits it to only accepting inputs between a single thumb and opposing finger pair, and modification to the system needs to be done to let it accept inputs from different thumb and opposing finger pairs. And finally, the limited number of source detector pairs used in fNIRS data acquisition may not be enough to cover the whole motor area region of the brain.

To address these limitations, a long-term follow-up study containing training sessions, adjustable difficulty levels, modified experiment block design, and so forth, can be performed to give more insight regarding the effectiveness of adding a force control component to rehabilitation outcomes of VR-based programs.

### Conclusions

In this study, the effects between the regular hand-tracking system of VR head-mounted displays and the novel VR input system on brain activity were compared. To the author’s best knowledge, this is the first system specifically designed for training or rehabilitation in the literature to incorporate a force control component to a VR hand-tracking system. The fNIRS results from this study suggest that the addition of a force control component to VR hand-tracking systems could make them more effective for training or rehabilitation by inducing greater and more widespread activity in the cortex, thus making it better for promoting neural plasticity. Additionally, the results from this study can be used as a reference for the development of new VR-based training or rehabilitation systems, which incorporate force control as a gameplay component.

## Abbreviations

ADL: activity of daily living
COV: coefficient of variation
CR: connection ratio
CS: connection strength
fMRI: functional magnetic resonance imaging
fNIRS: functional near-infrared spectroscopy
FSR: force-sensitive resistor
HbO: oxygenated hemoglobin
HbR: deoxygenated hemoglobin
HRF: hemodynamic response function
lM1: left primary motor cortex
M1: primary motor cortex
MVC: maximum voluntary contraction
NCKU: National Cheng Kung University
PFC: prefrontal cortex
PMC: premotor cortex
rM1: right primary motor cortex
ROI: region of interest
rPFC: right prefrontal cortex
rPMC: right premotor cortex
rSMA: right supplementary motor area
SMA: supplementary motor area
STROBE: Strengthening the Reporting of Observational Studies in Epidemiology
VR: virtual reality
wFC: with force control input system
woFC: without force control input system
